# Capillary-Mitochondrial Oxygen Transport in Muscle: Paradigm Shifts

**DOI:** 10.1093/function/zqad013

**Published:** 2023-03-16

**Authors:** David C Poole, Timothy I Musch

**Affiliations:** Departments of Kinesiology, Anatomy and Physiology, College of Veterinary Medicine, Kansas State University, Manhattan Kansas 66506, USA; Departments of Kinesiology, Anatomy and Physiology, College of Veterinary Medicine, Kansas State University, Manhattan Kansas 66506, USA

**Keywords:** exercise intolerance, capillary hemodynamics, oxygen transport, oxygen uptake kinetics, heart failure, muscle blood flow

## Abstract

When exercising humans increase their oxygen uptake (V̇O_2_) 20-fold above rest the numbers are staggering: Each minute the O_2_ transport system - lungs, cardiovascular, active muscles – transports and utilizes 161 sextillion (10 ^21^) O_2_ molecules. Leg extension exercise increases the quadriceps muscles’ blood flow 100-times; transporting 17 sextillion O_2_ molecules per kilogram per minute from microcirculation (capillaries) to mitochondria powering their cellular energetics. Within these muscles, the capillary network constitutes a prodigious blood-tissue interface essential to exchange O_2_ and carbon dioxide requisite for muscle function. In disease, microcirculatory dysfunction underlies the pathophysiology of heart failure, diabetes, hypertension, pulmonary disease, sepsis, stroke and senile dementia. Effective therapeutic countermeasure design demands knowledge of microvascular/capillary function in health to recognize and combat pathological dysfunction. Dated concepts of skeletal muscle capillary (from the Latin *capillus* meaning ‘hair’) function prevail despite rigorous data-supported contemporary models; hindering progress in the field for future and current students, researchers and clinicians. Following closely the 100th anniversary of August Krogh’s 1920 Nobel Prize for capillary function this Evidence Review presents an anatomical and physiological development of this dynamic field: Constructing a scientifically defensible platform for our current understanding of microcirculatory physiological function in supporting blood-mitochondrial O_2_ transport. New developments include: 1. Putative roles of red blood cell aquaporin and rhesus channels in determining tissue O_2_ diffusion. 2. Recent discoveries regarding intramyocyte O_2_ transport. 3. Developing a comprehensive capillary functional model for muscle O_2_ delivery-to-V̇O_2_ matching. 4. Use of kinetics analysis to discriminate control mechanisms from collateral or pathological phenomena.

## Introduction: What Must Capillaries Do?

Breaking World athletics records and achieving monumental physical milestones (4 min mile, 2 h marathon) captivates public attention whilst extending the proven boundaries of physical performance. Development of novel methods (e.g., near infrared spectroscopy, NIRS; nuclear magnetic resonance, NMR) combined with high fidelity measurements (spatial and temporal) mathematical and kinetics modeling approaches have better resolved the physiology of elite performance in health and also our understanding of the exercise intolerance that erodes life quality and predicates morbidity and mortality in disease.

**Figure 1.**
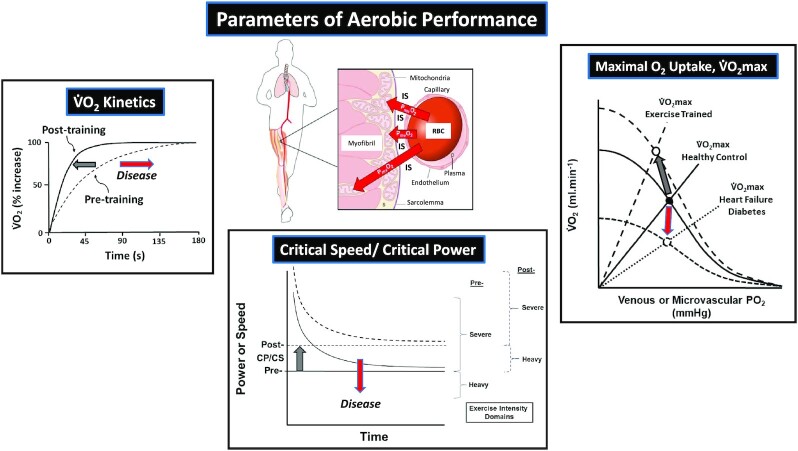
Principal parameters of aerobic performance that determine exercise capacity and their plasticity: (Left) O_2_ uptake (V̇O_2_) kinetics indicate the speed of V̇O_2_ increase following the onset of exercise. Dashed line pretraining, solid line posttraining. (Centre) Critical Power (CP) or Speed (CS) (asymptote of the Power/Speed—Time relation during high intensity exhausting exercise). Solid lines pretraining, dashed lines posttraining. (Right) Maximal V̇O_2_ (V̇O_2_max, symbols) conflates perfusive (curves from ordinate) with diffusive O_2_ conductance (straight lines from origin). Solid line is pretraining, upper dashed lines are posttraining and lower small dashes indicating disease. Direction of change indicated by gray (training) and red (disease, aging) arrows. This *Evidence Review* addresses the fundamental mechanisms by which O_2_ molecules transit the blood-mitochondrial space to power muscle contractile function (*inset*). RBC, red blood cell; IS, interstitial space; and P*mv*O_2_, microvascular partial pressure of O_2_.

With these developments, iconic perspectives of skeletal muscle structure and functional control have been overturned, especially as regards O_2_ delivery (Q̇O_2_) and its instantaneous matching to V̇O_2_. We are far closer to understanding what the key functional parameters of sustained muscular performance are—V̇O_2_ kinetics, critical power (CP or speed, CS), and the maximum V̇O_2_ (V̇O_2_max) (Figure 1). A compelling weight of evidence supports that the speed of V̇O_2_ kinetics in healthy young individuals is controlled by mitochondrial energetics rather than Q̇O_2_^rev.^^1^ and, in part, by constraining the O_2_ deficit fast V̇O_2_ kinetics facilitate a greater CP.^2^ CP, which is sensitive to muscle Q̇O_2_^3–6^ designates the boundary between the heavy and severe exercise intensity domains and, therefore, sustainable from nonsustainable exercise.^7,8^ That experimentally elevating muscle Q̇O_2_, by raising arterial hematocrit ^rev.^^9^ or inspired O_2_ fraction,^10^ increases V̇O_2_max demonstrates that Q̇O_2_ limits V̇O_2_max.

Crucially, any model of muscle microcirculatory control and function must cohere with and support these known facets of oxidative performance. Specifically, resting muscle preparations cannot have a V̇O_2_ that is Q̇O_2_ dependent (see ^10–12^; c.f. ^13^), during exercise V̇O_2_ and blood flow (Q̇) should increase in a ratio of ∼1:5 or 6,^14–16^ across a range of metabolic rates venous O_2_ content should fall (and fractional extraction i.e., arterial–venous O_2_ content, rise) as a hyperbolic function of increasing V̇O_2_.^14,16^ Moreover, when measured within the skeletal muscle, V̇O_2_ should increase with little delay in an exponential fashion with a time constant (τ) of some 10 to 40 s in young, healthy individuals.^17–20^^; rev.^^1^

The most pervasive capillary control models over the past century are based upon August Krogh’s Nobel prizewinning “capillary motor” schema.^21–25^ Therein the vast majority of capillaries are, via a contractile endothelium (now debunked) or pericyte constriction (see Role of Pericytes in Controlling Skeletal Muscle Capillary Hemodynamics below), closed in resting muscle but open up and support red blood cell (RBC) flux during muscle contractions. In this review we will demonstrate, across a range of physiological stimuli, that this schema cannot explain the observed behavior, either temporally (i.e., speed of V̇O_2_ kinetics) or quantitatively (i.e., CP, V̇O_2_max), and is unnecessary to support the dynamic control of microcirculatory Q̇O_2_ and V̇O_2_ kinetics.

Moreover, beyond the Kroghian binary concept of capillaries (i.e., open with a fixed O_2_ delivery or closed), our understanding of the transport of O_2_ from RBC to mitochondria has undergone substantive revision based upon recent discoveries. For instance, the diffusivity of O_2_ across its “final frontier” is likely dependent, in part, upon: (1) Newly discovered channels in the RBC membrane. (2) Recruitment of capillary exchange surface *along the capillary length* (longitudinal recruitment). (3) Network interdependence of O_2_ exchange among adjacent microvessels. (4) More homogeneous O_2_ pressures in the interstitial space surrounding myocytes. (5) O_2_ tracking along sarcolemmal and intracellular membranes, including interconnected mitochondria. (6) Interplay between deoxygenated myoglobin (Mb) and nitric oxide (NO) release to control cytochrome oxidase activity and better spatially coordinate mitochondrial ATP production and O_2_ availability. These considerations are important for construction of a contemporary model of capillary function and O_2_ transport: One that has far less reliance on control at the individual capillary level and, at least in health, minimizes the importance of capillary–mitochondrial diffusion distances per se.

### A Brief History of Capillaries

When William Harvey’s *De Motu Cordis* was published in 1628^26^ there was no concept of capillaries. Rather, blood was supposed to flow from the right to left heart through the pores of Galen in the interventricular septum. Some years later, after boiling organs, including kidneys, liver, lungs, and spleen and examining them closely, Harvey described “*capillamenta*” which must have been small arteries and veins or arterioles/venules as capillaries cannot be resolved by the naked eye.^27^ He thus concluded that, in the periphery, blood flows: “…*from the arteries into the veins either directly by means of anastomosis or indirectly through the porosities of the flesh*…” ^27^^, p. 88^ Had Harvey been cognizant, at that time, of the eminent Islamic scholar known as Ibn al-Nafis (1213 to 1288, full name: Ala al-Din Abu al-Hassan Ali Ibn Abi-Hazm al-Qarshi al-Dimashq), whose *Commentary on Anatomy in Avicenna’s Canon* became known to the Western world only in the early 20th century, he may have reconsidered his “*porosities of the flesh*” concept. As translated from “*Commentaries*…” by Meyerhof^28^” “…*there is no passage between these two cavities [right and left ventricles]; for the substance of the heart is solid…*” and “….*the penetration of the blood into the left ventricle is from the lung*…” leading Ibn al-Nafis to conclude that there must be small communications between the pulmonary artery and vein. See West,^29^ for a reproduction of the original Arabic text opposing the presence of septal connections between the ventricles from Meyerhof.^28^ Thus, at least as early as the 13th century in the Arab world the pores of Galen in the interventricular septum had been falsified. In Europe, Michael Servetus (1511 to 1553), who, West^29^ speculates, may have been aware of Ibn al-Nafis’s work—though historians consider this unlikely—wrote that the blood passed from the pulmonary artery through the lung to the left ventricle and became “*reddish-yellow*” in the process.^30^^; see also^^29^ As a mark of Harvey’s intuitive brilliance, a few years before his death in 1657, he experimented with the pulmonary circulation of a throttled man. After ligating certain vessels he demonstrated that water flowed easily from the pulmonary artery through the lungs and into the left ventricle.^31^^; rev.^^29^ A finding that, for the emerging generations of microscopists, identified the lungs as an organ where visualization of the smallest vessels connecting the arterial and venous circulation might be possible in vivo.

Enter Marcello Malpighi who was born serendipitously in 1628, the year Harvey published *De Motu*, in Crevalcore near Bologna. Malpighi used both single magnifying lenses and a compound double-convex lens microscope and observed capillaries in the alveolar walls of the frog lung.^32^ He described the capillaries as “*tortuous*” and clarified conclusively that blood “*is always passed through tubules*” remaining within the vessels and not “*poured out into spaces*” (translation from^33^). Whereas, Jan Swammerdam (1637 to 1680), had described RBCs or corpuscles three years earlier;^34^^, see also^^35^ Malpighi mentioned these only as “*particles*” within the blood, probably because he could not determine their color.^36^

Stephen Hales (1677 to 1761), minister of Teddington parish near London, may have been the first person to coin the term “capillary.”^37,38^ Hales estimated the pulmonary capillary diameter to be around 17 µm (i.e., ∼2- to 3-fold the average diameter of muscle capillaries) with pulsatile flow and a RBC transit time just over 2 s. Capillaries were distinguished from other blood vessels by Marshall Hall (1790 to 1857) as being of “…*an intermediate station…*” interposed between the “…*last branches of the arterial system and the first roots of the venous*…” ^39^^, p.^^18^ He noted that, within capillaries compared with upstream vessels, blood velocity was halved and intuited that “…*a more diffused and slower circulation is required for administering to the nutrient…functions”* and considered capillaries to be of “*uniform character and dimensions*.”^39^^, pp.^^29–30^ Johannes Müller (1801 to 1858), in 1843 reinforced the notion that capillaries were “*the same diameter throughout*”^40^ but the field was obscured with confusing terms such as “capillary arteries,” “capillary veins”, and “minute vessels.” ^41^ Improvements in light microscopy toward the middle of the 19th century revealed the cell as the primary structural base of tissues. ^42,43^^; rev.^^44^ This realization and Schwann’s “cell theory” were focal to the recognition of the capillary wall or endothelium.

Reminiscent of Harvey, J.W. Earle in 1835 portrayed capillaries as membrane-less channels such that: “…*blood in the finest capillaries…flows…in simple furrows, or canals, whose walls are formed by the surrounding cellular tissue*.” ^45^^, p.^^8^ At that time it was considered impossible for “*the processes of nutrition and absorption being carried on through the coats of vessels*.”^42^ Counter to this notion was that capillaries in the ears of reptiles and birds when injected with dyes were distinct from adjacent tissues—evidencing “parieties” or walls.^46^ It was left to Theodor Schwann (1810 to 1882), in 1839 to identify what would later be called the endothelium in the tails of tadpoles.^43^ However, adherents to each side of the argument, walls *versus* no walls, engaged in strident debate based upon assumed properties of such a barrier and the presumption that it would prevent the known blood-tissue transit of, for example, leukocytes (see ref.^44^ for a stimulating account of the controversy). This despite Augustus Waller’s (1814 to 1870) demonstration, in real time, of leukocytes “*protruding*” from vessels at the site of inflammation in his frog’s tongue intravital microscopy preparation.^47^ A process called diapedesis.

At the close of the 19th century Julius Cohnheim (1839 to 1884), a student of Rudolf Virchow (1821 to 1902), had demonstrated that the capillary wall was a living organ rather than simply an inert membrane.^48^ This set the stage for addressing how capillaries subserve their cardinal responsibility for facilitating blood–tissue exchange. Ernest Henry Starling (1866 to 1927) would take up the challenge for substrate and fluid exchange, whereas Schack August Steenberg Krogh’s (1874 to 1949) focus was O_2_ delivery, primarily in skeletal muscle.

Since Krogh’s 1920 Nobel prize, the dominant concepts of capillary function, especially within skeletal muscle, have been driven by Krogh’s work and theories.^21–25^ This despite a more recent compelling weight of opposing evidence garnered with advanced technologies under rigorously controlled cardiovascular conditions. Major Kroghian concepts include: (A) Most capillaries being “*closed*” in resting muscle and opened or “*recruited*” at the onset of contractions. (B) Each capillary representing an independent fixed unit of O_2_ delivery with intracapillary diffusion distances determining the efficacy of mitochondrial O_2_ delivery. Despite these notions being resoundingly falsified, they remain at the forefront of undergraduate and medical school teaching and exert a persistent influence over the microcirculatory control field today.

**Figure 2.**
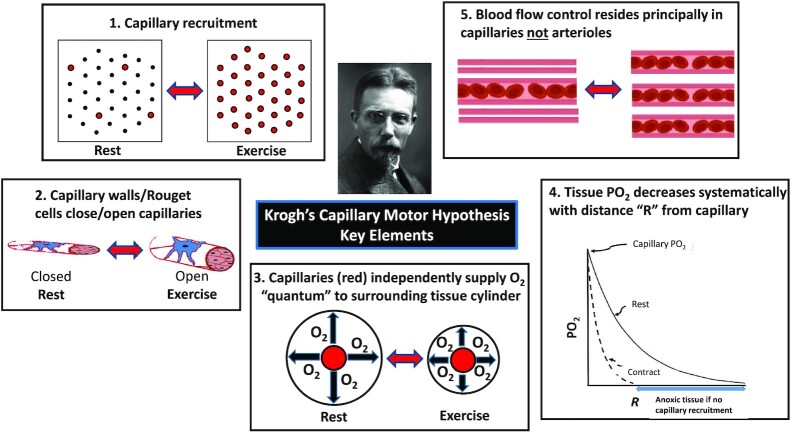
Primary tenets of August Krogh’s capillary motor hypothesis.^21–23^ Anticlockwise from top left: (1) Most capillaries do not support red blood cell (RBC) flux in muscle at rest and are “recruited” with the onset of contractions. (2) At rest either contractile walls or Rouget cells (now called pericytes) close most capillaries and relax during muscle contractions to facilitate RBC flux. (3) Capillaries independently provide a fixed RBC flux to surrounding Krogh cylinder of tissue. With capillary recruitment that cylinder decreases in volume. (4) Krogh–Erlang model estimates O_2_ partial pressure [PO_2_] at distance “R” from capillary. Without capillary recruitment muscle regions (blue) would become anoxic. (5) In this schema, capillaries constitute an important locus of blood flow control. See text (*Myths #1 to 8*) for more details.

In many ways, the microcirculation today is treated as a classic—“*everybody talks about it but nobody reads it*” to paraphrase the great Mark Twain.^49^ For instance, one of the most memorable and enduring “facts” regarding the human circulation is that the total length of microvessels in skeletal muscle (or the human body) is 100 000 km or more; sufficient to encircle the Earth at the equator about 2.5 times. This figure comes initially from Krogh’s book,^25^^, p.^^10^ where capillarity values from guinea pigs are inappropriately applied to human muscles, and is grossly in error. Our best estimates from contemporary data yield a total length of some 9 000 to 19 000 km; less memorable, perhaps, but more accurate and supporting a very different regulation of capillary structure and function in health and disease. Specifically, where the blood volume necessary to fully perfuse the muscle capillary bed is only 2% to 3% of total blood volume versus over 30% (Krogh’s estimate). In the latter instance, if correct, poorly regulated (muscle) capillary opening and perfusion could compromise cardiovascular regulation and homeostasis. Figure 2 portrays some of the essential elements from Krogh, many of which are still retained in the microcirculation lexicon today.

**Figure 3.**
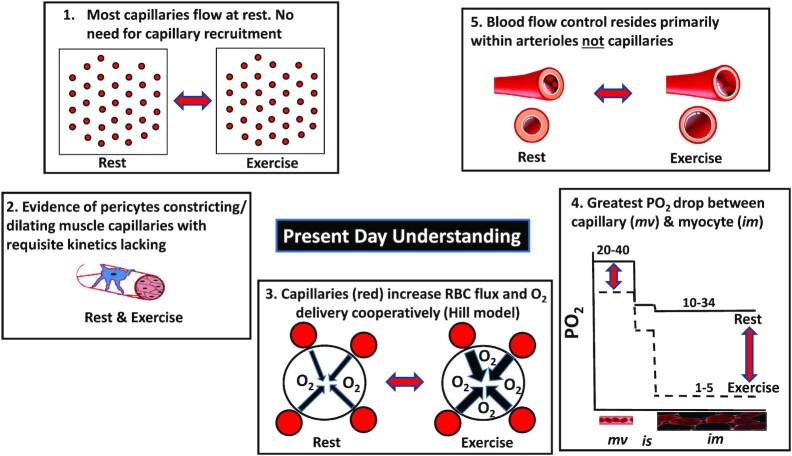
Present day understanding revises Figure 2 premises as follows: (1) Most capillaries support RBC flux at rest and so there is not much scope for de novo capillary recruitment. (2) Evidence of either contractile endothelium or pericytes closing capillary lumen and opening during contractions with requisite speed lacking. (3) Hill model incorporating capillary geometry and, possibly, interstitial space, allows cooperative O_2_ delivery among capillaries (and other vessels) and reduces supplied tissue volume with distance from capillary. (4) Most of the partial pressure (PO_2_) drop between red blood cell in the microvasculature (*mv*), across the interstitial space (*is*) and mitochondria occurs very close to the capillary such that intramyocyte (*im*) PO_2_ during contractions is extremely low. (5) It is now recognized that the majority of vascular resistance and thus blood flow control resides at the arteriolar level. See text (*Myths #1 to 8*) for more details.

**Figure 4.**
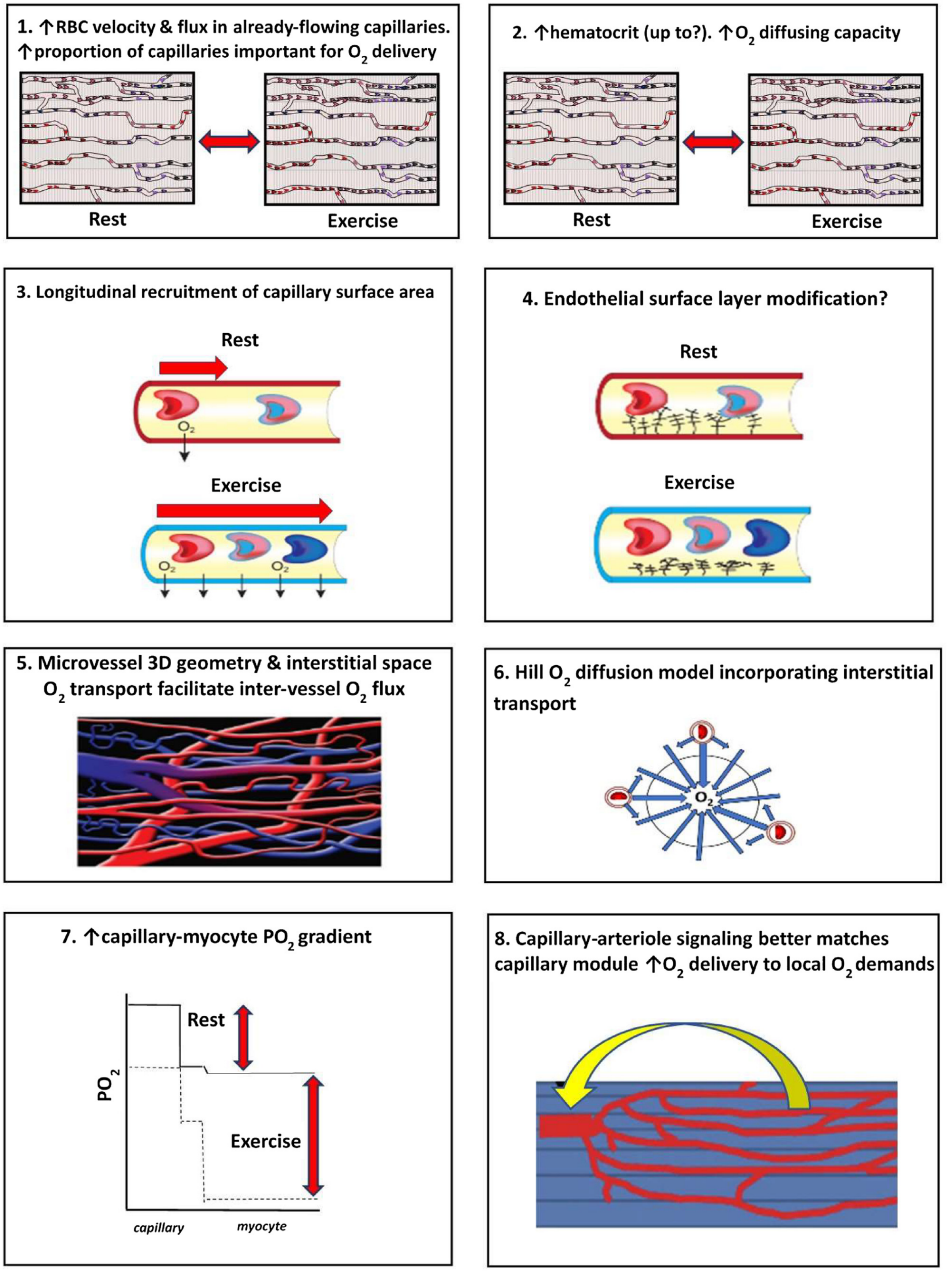
A contemporary model of the muscle microcirculation: (1) At exercise onset, capillaries are not recruited de novo but red blood cell (RBC) velocity and flux increase and raise the proportion of capillaries important for O_2_ delivery. If mean muscle blood flow increases by 3-fold for moderate/light intensity exercise and 30-fold for heavy/severe intensity exercise, assuming a normal distribution of capillary RBC fluxes at rest and during contractions, the percentage of capillaries having an RBC flux below 10 cells/s (resting data^114^; contracting^89^) would decrease from ∼20%–30% to ∼10% and to ∼1-2%, respectively. (2) Elevated capillary hematocrit (∼30%; rat intravital microscopy^89^; human NIRS^243^) increases O_2_ diffusing capacity commensurately. (3) Increased functional surface area is recruited along the length of capillaries (so-called longitudinal recruitment). (4) There may be a modification of the endothelial surface layer (glycocalyx) that facilitates the higher hematocrit. This represents an exciting current field of investigation. (5) The 3D geometry of the microvascular bed facilitates between-vessel O_2_ exchange by means of the interstitial space. (6) The Hill model better represents capillary-myocyte O_2_ transport than the Krogh model (pinpoint O_2_ delivery). (7) Despite that microvascular PO_2_ falls during exercise (reflecting the greater fractional O_2_ extraction) the precipitous drop in intramyocyte PO_2_ means that the overall PO_2_ gradient between RBC and intramyocyte space actually increases from rest to exercise. (8) Upstream signaling from capillaries (and also potentially venules) vasodilates the terminal arterioles specific to individual capillary modules. This mechanism and potentially microvascular network effects are likely key to facilitating O_2_ delivery-O_2_ utilization matching across the exercising muscle.

The following section (*Pervasive Myths and The Microcirculation*) presents some of the major time-honored beliefs originating from Krogh;^21–25^ (see Figure 2) and Chambers and Zweifach, as regards so-called precapillary sphincters^50^ and, in opposition, the more recent evidence that informs and shapes a contemporary understanding of capillary function and blood-mitochondrial O_2_ transport matching structure and function to physiology (Figures 3 and 4).

### Pervasive Myths and the Microcirculation

Myth 1: *“Capillary recruitment”: In resting skeletal muscle(s) most capillaries are closed and do not support RBC or plasma flux. At the start of contractions, these capillaries are recruited and support RBC flux. Therefore an increase in the number of RBC-flowing capillaries is requisite to support V̇O_2_ kinetics and elevated blood-tissue O_2_ flux*.

#### Evidence Falsifying Myth 1

When circulation-intact resting muscles are visualized by intravital microscopy under physiological conditions (i.e., normotensive, nonhyperoxic, or pharmacologically vasoconstricted, not over stretched or surgically damaged) the vast majority (i.e., >80%) of capillaries support RBC flux (see Figure 3 #1 and Table 1, ^51–106^ right side^72–106^). Moreover, that minority of capillaries not supporting RBC flux are “open” and not collapsed. Thus, following the onset of contractions increased perfusive (RBC flux) and diffusive (DO_2_, hematocrit) O_2_ transport principally occurs via elevated RBC flux, velocity and hematocrit in already-flowing capillaries (Figure 4, #s 1 and 2). As capillary hematocrit at rest is only ∼15% to 20%, on average, increases toward systemic values (i.e., ∼45%) can substantially increase capillary RBC content and therefore DO_2_.^107,108,109^ As RBC velocity increases in concert with elevated fractional O_2_ extraction—from ∼25% at rest to as much as ∼90% during contractions—capillary exchange surface area is recruited along the length of individual capillaries, a process known as “*longitudinal recruitment*,” which contributes to the elevated DO_2_ (Figure 4, #3).^110^ An important scientific goal will be to apportion the substantial increase in DO_2_ from rest-contractions among microvascular (e.g, RBC aquaporin + rhesus channels, hematocrit, RBC distribution [relative to fiber(s) V̇O_2_], longitudinal recruitment, network effects), and myocyte-associated processes (interstitial O_2_ diffusion pathways, membrane O_2_ transport [sarcolemma, mitochondrial membranes], Mb, simple diffusion). These latter processes are considered below (see the section “Intramyocyte O_2_ Transport: Sarcolemma-Mitochondria”).

**Table 1.** Research papers supporting capillary recruitment (left column, most capillaries not flowing) and those demonstrating that most capillaries support red blood cell flux in resting skeletal muscle (right column) and are, therefore, not recruited during contractions.

KROGHIAN: OPENING OF PREVIOUSLY “CLOSED” CAPILLARIES (supports capillary recruitment)	CONTEMPORARY: MOST CAPILLARIES SUPPORT RBC AND/OR PLASMA FLOW AT REST (does not support capillary recruitment)
Krogh, 1919a to c (21 to 23) (assorted muscles/species)	Eriksson and Myrhage, 1972 (72) (cat, tenuissimus)
Wagner and Latham, 1975 (51) (lung)	Burton and Johnson, 1972 (73) (cat, sartorius)
Gorczynski and Duling, 1978 (52) (hamster, cremaster)	Klitzman and Duling, 1979 (74) (hamster, cremaster)
Honig et al. 1980, 1982 (53,54) (dog, gracilis, indirect)	Klitzman et al. 1982 (75) (hamster, cremaster)
Gray et al. 1983 (55) (chicken, latissimus dorsi)	Renkin et al. 1981 (76) (rabbit, leg muscles)
*Rattigan et al. 1997ab (56,57) (rat, hindlimb muscles, indirect)	Hudlicka et al. 1982 (77) (rat, EDL)
Fuglevand and Segal, 1997 (58) (computer model)	Vetterlein et al. 1982 (78) (rat, myocardium)
Parthasarathi and Lipowsky, 1999 (59) (rat, cremaster, nonphysiologic PO_2_’s)	Kayar and Banchero, 1985 (79) (rat, gastrocnemius, in vivo)
*Rattigan et al. 2001 (60) (rat, hindlimb muscles, indirect)	Snyder et al. 1992 (80) (rat, in vivo, various muscles)
*Youd et al. 1999,2000 (61,62) *(rat muscles, indirect, 1-MX)	Hudlicka, 1985 (81) (review)
*Zhang et al. 2004 (63) (1-MX, CEU)	Tyml, 1986 (82) (frog, sartorius)
*Vincent et al. 2004, 2006 (64,65)	Oude Vrielink et al. 1987 (83) (rabbit tenuissimus)
*Wheatley et al. 2004 (66)	Bosman et al. 1995 (84) (rabbit, tenuissimus)
*Rattigan et al. 2003, 2005 (67,68)	Poole et al. 1997 (85) (rat, spinotrapezius)
*Clark et al. 2008ab (review) (69,70)	Kindig and Poole, 1998 (86) (rat, diaphragm)
Fry et al. 2013 (71) (hamster, cremaster, modeling)	Kindig and Poole, 1999 (87) (rat, spinotrapezius)
	Kindig and Poole, 2001 (88) (rat spinotrapezius)
	Kindig et al. 2002 (89) (rat, spinotrapezius)
	Richardson et al. 2003 (90) (rat, spinotrapezius)
	Ellis et al. 2002 (91) (direct, rat EDL)
	Padilla et al. 2006 (92) (rat, spinotrapezius)
	Copp et al. 2009 (93) (rat, spinotrapezius)
	Fraser et al. 2012a (94) (rat, EDL)
	Fraser et al. 2012b,2013 (95,96) (modeled on rat EDL)
	Bateman et al. 2015 (97) (rat hindlimb muscles)
	McClatchley et al. 2019 (98) (mouse gastrocnemius)
	Akerstrom et al. 2020 (99) (rat EDL)
	McClatchley et al. 2020 (100) (mouse gastrocnemius)
	Hirai et al. 2021 (101) (rat, spinotrapezius)
	Mendelson et al. 2021 (102) (rat, EDL)
	Weber et al. 2022 (103) (rat, spinotrapezius)
	Horn et al. 2022 (104) (rat, spinotrapezius)
	Schulze et al. 2022 (105) (rat, spinotrapezius)
	Mendelson et al. 2022 (106) (rat, EDL)

The propensity of papers demonstrating RBC and/or plasma flow in most capillaries in resting muscle (right side) are either direct observations of muscle microcirculation or studies using endothelial staining of capillaries. Unlike those on the right side, many of those on the left side use indirect methods such as 1-MX (1-methyl xanthine) or contrast-enhanced ultrasound (CEU). Updated from Poole el al.^107^ See text for more details.

Using a combination of intravital microscopy and phosphorescence quenching in the rat spinotrapezius muscle, rapid, and physiological V̇O_2_ kinetics (time constant ∼30 s) have been demonstrated in the absence of any increase in the numbers of RBC-perfused capillaries.^20,89^ This profile matches closely that measured for pulmonary^17^^; rev.^^1^ and leg muscles^19,111,112^ V̇O_2_ in humans during voluntary exercise.

A valid concern reflects that, because direct observation of the skeletal muscle capillary bed requires that the animal be anesthetized, the presence of RBC flux in most capillaries may be an artifact of anesthesia or surgery. However, several lines of evidence support that neither anesthesia nor surgery perturb the underlying physiology in this regard. Specifically: (A) Surgical exteriorization does not alter muscle Q̇^113^ nor impact the fundamental proportionality between muscle V̇O_2_ and Q̇.^16^ (B) Using endothelial dye perfusions essentially *all* capillaries in muscles of anesthetized and conscious rats at rest evidence flow (at least plasma) nullifying the opportunity for de novo recruitment.^79,80^ (C) Broadly accepted values for resting muscle blood flows are compatible with 80% to 90% of total capillaries sustaining flow with >10 RBCs/s.^89^,^114–119^ (D) Total quadriceps (haemoglobin + myoglobin), measured using near-infrared spectroscopy (NIRS), increases <30% from rest-exercise.^120–122^^, rev.^^119^, ^123–125^ This observation is inconsistent with the substantive recruitment of previously “closed” capillaries.

One theoretical argument advanced to support that capillary recruitment must occur from rest-exercise is that this is necessary to limit the fall in capillary RBC transit times. However, this notion is specious because, irrespective of how you get there—recruitment or simply increased flux in already flowing capillaries—RBC transit time during maximal exercise will be exactly the same as it is determined by the ratio of capillary volume/blood flow. Moreover, the estimations of Richardson and colleagues,^126,127^ based upon some of the highest muscle blood flows measured in human knee-extensors, support that capillary RBC mean transit times of only ∼0.1 s can facilitate fractional O_2_ extractions as high as 80%.

Myth 2: *Capillaries have a binary function: Either closed or open (see* *Figure 2* *#2) with the latter supporting a fixed capacity (i.e., same at rest and contractions when open) for RBC flux and O_2_ delivery*.

#### Evidence Falsifying Myth 2

At rest and during contractions there is a pronounced heterogeneity of RBC flux, velocity, and hematocrit among capillaries. This is true even for adjacent capillaries served by the same terminal arteriole. If there is little or no de novo recruitment of flowing capillaries from rest to contractions then the perfusive (Q̇O_2_) and diffusive (DO_2_) capacity of individual capillaries (see ^89^ and Table 1, right side for references) must increase many fold to support as much as 100-fold elevation of muscle V̇O_2_.^126^ Conceptually it is important to recognize that some capillaries at rest have such a low RBC flux and/or hematocrit that they do not contribute substantially to support metabolism. During contractions, however, their increased RBC flux and hematocrit facilitate a substantial contribution of these capillaries to muscle V̇O_2_ (Figure 4 #1).

Myth 3: *Capillaries are straight, unbranched vessels that supply O_2_ independent of all others to a cylinder of surrounding tissue*.

#### Evidence Falsifying Myth 3

**Figure 5.**
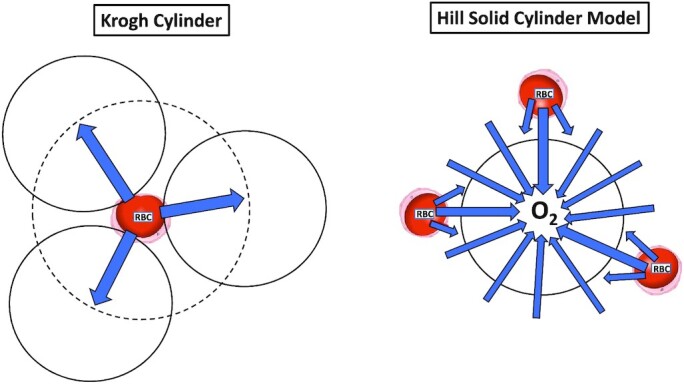
The Hill solid cylinder model provides a far more efficient O_2_ delivery to the myocyte (black circle) as the O_2_ path is not restricted to the small physical space apposed to the red blood cell (RBC). Thus, for the Hill model the O_2_ flux density—and therefore the apparent resistance to O_2_ diffusion into the myocyte—is far less.^130^

Capillaries evidence substantial tortuosity, especially at short muscle sarcomere lengths,^128,129^ and are highly interconnected by branches (Figure 4 #5).^128^ This geometry serves to increase muscle capillary volume and surface area and the proportion of myocyte surface immediately adjacent to RBCs within capillaries. Thus, the Hill cylindrical model of O_2_ diffusion is likely more appropriate than the “pinpoint” O_2_ source that Krogh considered (cf. Figure 2 #3 with Figure 3 #3 and Figure 4 #6).^130^ Moreover, based upon measurements of interstitial (P*is*O_2_) that are far higher than intramyocyte (P*im*O_2_) PO_2_’s (Figure 3 #4;^131–134^^; rev.^^135^ see also Intramyocyte O*_2_*Transport: Sarcolemma-Mitochondria subsection below) and O_2_ diffusion among different vessels in the microcirculatory network,^136,137^ the latest O_2_ transport models advocate for dependent tissue volumes that *decrease* rather than increase (see Krogh–Erlang model) with distance from the O_2_ source—be that the capillaries or interstitial space (seeFigure 3 #3; Figure 4 #6, and Figure 5).^107,124,125^

Myth 4: *According to the Krogh–Erlang model O_2_ partial pressure (PO_2_) decreases systematically with distance from the capillary generating anoxic loci in resting muscle beyond the boundary of the “Krogh cylinder” (* *Figure 2* *#4). If true this places an obligatory limitation on capillary–mitochondrial diffusion distances*.

#### Evidence Falsifying Myth 4

Measurements of microvascular PO_2_ (P*mv*O_2_), P*is*O_2_ and P*im*O_2_ at rest and during contractions, not technically feasible in Krogh’s time, have revealed very different profiles than hypothesized by the Krogh–Erlang model (cf. Figure 2 #4 and Figure 3 #4, Figure 4 #7). Specifically, P*mv*O_2_ is regulated as a function of muscle fiber type, being significantly higher at rest and during contractions in slow than fast twitch muscles. ^138,139^ PO_2_ falls steeply across the capillary endothelium being some 8 to 12 mmHg lower for P*is*O_2_ than P*mv*O_2_.^133^ It has not been ruled out that O_2_ consumption by the frequency domain phosphorescence method might contribute to some of this gradient. However, the relative constancy of the gradient from rest to contractions, and the very low potential for this method to consume appreciable O_2_, at least in comparison to the extant V̇O_2_ of the tissue, support that this microvascular-interstitial gradient is not an artefact of the technique. Thus, because P*im*O_2_ during contractions decreases to as low as 1 to 5 mmHg (data from dog gracilis^134,135^) and human quadriceps^140–143^) there exists an appreciable (i.e., several mmHg) trans-sarcolemmal (i.e., P*is*O_2_ to P*im*O_2_) PO_2_ gradient during contractions. Although possibly a limitation of the cryomicrospectroscopy and proton NMRS measurement techniques neither radial nor longitudinal intracellular P*im*O_2_ gradients or anoxic loci (i.e., 0 mmHg) have been detected. If intramyocyte PO_2_ gradients do exist, because of the overall low P*im*O_2_, they must be small. These data support the notion that intramyocyte O_2_ transport is extremely effective and therefore that diffusion distances from sarcolemma to even the most distant part of the mitochondrial reticulum are not limiting (see also Skeletal muscle Mitochondria: Structure-Function and Relevance….below).

Myth 5: *RBC and plasma flow through capillaries are controlled by so called precapillary sphincters*.

#### Evidence Falsifying Myth 5

Krogh’s “*capillary motor regulating mechanism*”^21–23^,^144^ that won him the Nobel prize was central to the capillary recruitment hypothesis and emplaced capillaries themselves in control of muscle Q̇ (Figure 2 #5). Krogh initially considered that the capillary endothelium was itself contractile—as conveyed in his Nobel lecture drawing of a “capillary” that becomes vastly dilated (>50 µm diameter) when mechanically irritated.^24,25^^; rev.^^125^ However, subsequent experiments across tissues from different species led his focus to Rouget cells^145, 146^—now called pericytes—as the source of unitary capillary constriction and dilation (see Schmidt-Nielsen, 1984 for her fascinating account of these experiments).^147^ Despite intense investigation, initially by Bjovulf J. Vimtrup^148,149^ over the ensuing decades, there was not incontrovertible evidence that pericytes could reversibly constrict (and close shut, Figure 2 #2) the capillary lumen (see ^125^ for a detailed account). Throughout the 1930s capillary contractility was repeatedly challenged with advocates^150^, ^151^ and opponents^152–156^ who regarded the capillary wall and associated structures as noncontractile.

In the absence of satisfactory evidence that either the capillary endothelium or pericytes could occlude the capillary lumen Benjamin Zweifach’s attention shifted to the beginning of the capillary, where the capillary branch departs from the arteriole. At that specific location he detected a narrowing which he termed a precapillary sphincter.^50^ Here the plot thickens. In the frog mesentery in 1937 Zweifach finds no precapillary sphincters,^155^ but in 1939 he found such in both mouse and rabbit mesentery.^156^ In 1942 Chambers and Zweifach produced motion pictures evincing “*sphincter like functioning of the precapillaries at their junction with the arteriole*.”^157^; see also Sakai and Hosoyamada.^158^ Despite Zweifach’s observations of precapillary sphincters solely in the mesentery and not in all species, within a decade, they were adopted as the presumptive site of capillary hemodynamic control within *ALL* microcirculations in physiological and medical textbooks.^159–165^^; rev.^^158^ Also disturbing, and a clear case of confirmation bias, was the propensity for researchers, from that point on, to attribute changes in vascular resistance to “*precapillary sphincter tone*” without any evidence for such.^166–168^ The extensive search for precapillary sphincters, anatomically and physiologically, detailed by Sakai and Hosoyamada,^158^ did not identify these structures in any tissue.

Given that absence of evidence is not necessarily evidence of absence with respect to structure it is also telling that presence of precapillary sphincters has not been demonstrated physiologically. For instance, hyperoxia, K_ATP_ channel blockade with glibenclamide and other conditions that decrease blood flow to skeletal muscles do so via arteriolar vasoconstriction and there is no evidence that RBC flux can be halted by active luminal constriction specifically in individual capillaries.^52^, ^169^^; rev.^^101,170^

Myth 6: *Following the onset of contractions it is necessary for muscles to deplete their O_2_ stores and produce vasoactive metabolites which then vasodilate arterioles and “open” up previously closed capillaries*.

#### Evidence Falsifying Myth 6

Advances in intravital microscopy, phosphorescence quenching measurement of PO_2_, ultrasound, NMR, and mathematical modeling have permitted resolution of rapid muscle metabolic and hemodynamic transient responses following exercise onset. These techniques have helped unveil the mechanistic bases for vascular control dynamics.

Logically, if the cardiovascular system is to subserve its primary function of blood pressure control, there must be a tight coordination between central cardiovascular (cardiac output) and muscle Q̇ dynamics. Skeletal muscle has such a great capacity for vasodilation that it has been described as the “*sleeping giant*” in that uncontrolled dilation would crash blood pressure catastrophically.^171^ Specifically, if muscle vasodilation occurred prior to cardiac output increasing, blood pressure would plummet. By the same token delayed muscle vasodilation would produce a potentially dangerous blood pressure spike. In young healthy subjects, neither extreme response is seen. Rather, following the onset of rhythmic cycling exercise mean arterial pressure rises modestly with response kinetics that are far slower than that of muscle Q̇ (i.e., time constant, τ, MAP, 89 s, muscle Q̇, 9 s):^172^ Supporting a close dynamic matching between the time course of both processes.

As early as 1895 the eminent Swedish physiologist Erik Johan Johansson (1862 to 1938) demonstrated that electrical stimulation of the legs of rabbits increased heart rate within 0.5 s.^173^ Later Krogh and Lindhard in 1913,^174^ measured correspondingly fast heart rate kinetics at the onset of cycle exercise in humans and Anrep and von Saalfeld in 1935 showed that muscle hyperemia was initiated simultaneously with the onset of contractions.^175^ Despite this evidence, as late as the close of the 20th Century, it was widely considered that, following the onset of exercise, “*muscle*” PO_2_ plummeted toward zero and subsequent vasodilation was dependent upon production of metabolites and sympathetic vasodilator activity.^176,177^

Today it is recognized that mechanisms initiating the increase of muscle Q̇ may be very different from those that sustain its exercising steady state.^178^^; rev.^^179^ Moreover, the obligatory role of the muscle pump^180,181^ in initiating the rapid hyperemia has been challenged by experiments supporting that contraction-induced vascular deformation induces an almost immediate vasodilation^182,183^ possibly involving integrins^184^ and mechanosensitive ion channels.^185^ Importantly, Behnke and Delp^186^ showed that isolated rat skeletal muscle arterioles do evince the requisite fast relaxation dynamics in response to elevated flow, acetyl choline and NO. However, Clifford and Tschakovsky,^179^ argued that a system which elevates muscle^19^,^181–183^,^187,188^ and capillary^89^ Q̇ within 1 s after a contraction is unlikely to depend on diffusion of some soluble vasoactive mediator. This conclusion would also be relevant to the extent to which sympatholysis^189^ could participate in the initial Q̇ response.^190^

**Figure 6.**
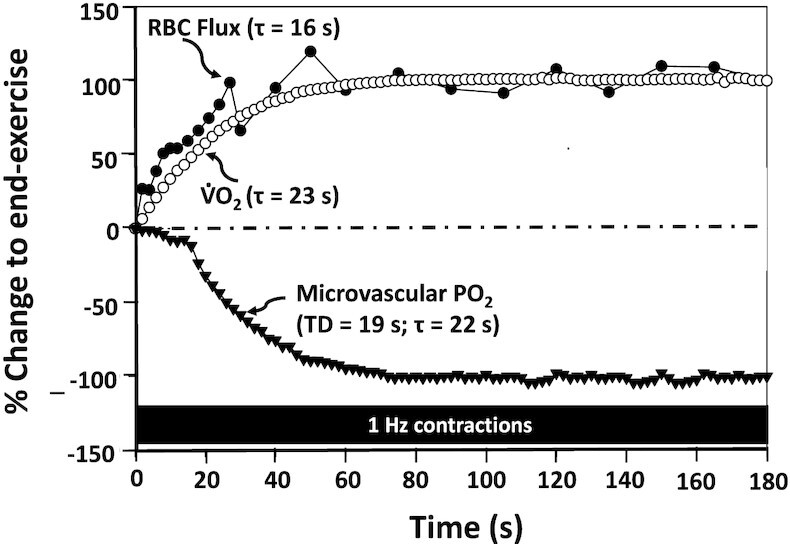
Temporal changes in capillary red blood cell (RBC) flux, oxygen uptake (V̇O_2_), and microvascular O_2_ partial pressure (P*mv*O_2_) following the onset of contractions in the rat spinotrapezius muscle (redrawn from ^20,89,192^). See text for more details.

Notwithstanding its precise mechanistic bases, following the onset of muscle contractions, in both exercising human muscle^19,111,112,191^ and capillary^20,89,192^ Q̇ increases as fast or faster than mitochondrial V̇O_2_ such that, for the first 10 to 20 s of contractions, muscle P*mv*O_2_ and venous O_2_ content do not fall and may even rise (Figure 6). ^19,20,89,192^^; rev.^^124^ Subsequently, as mitochondrial V̇O_2_ continues to increase and despite a continued increase of Q̇ to the steady-state or peak level, P*mv*O_2_ and venous O_2_ content decrease in a close-to-exponential fashion reflecting the rising overall V̇O_2_:Q̇O_2_ ratio.^16^ It is quite possible that, beyond the initial transient, further vasodilation and increased Q̇ reflects regional specific metabolic control that acts to enhance V̇O_2_-to-Q̇O_2_ matching and promotes greater fractional O_2_ extraction (see the section Modeling Capillary-Myocyte O_2_ Delivery below).

Myth 7: *Muscle O_2_ diffusing capacity (DO_2_) can only increase from rest to exercise by recruiting more capillaries and is determined principally by capillary density*.

#### Evidence Falsifying Myth 7

As seen above (see *Myth #1*), in healthy skeletal muscle, most capillaries support RBC flux and are, therefore, not available to be recruited. What can, and does, change from rest to contractions is the RBC flux and hematocrit within individual capillaries.^89^ This is crucial because O_2_ delivery models^108,109^ and experimental data^193^ support that the primary determinant of DO_2_ is the number of RBCs in the capillary bed, adjacent to the muscle fibers, within RBC-flowing capillaries. Accordingly, diseases such Type II diabetes^92^ and HF^90^ that decrease the proportion (and absolute number) of capillaries that support RBC flux, at least in animal models, are characterized by low DO_2_.^194^^; rev.^^195^ As discussed below in “*Oxygen transport from capillary to mitochondria*” the dependence of DO_2_ on capillary hematocrit may depend upon recently discovered aquaporin and rhesus channels in the RBC membrane.

The importance of knowing capillary RBC hemodynamics and not just capillary density per se was effectively demonstrated by Hepple and colleagues in the canine gastrocnemius-plantaris complex contracting at V̇O_2_max.^196^ Specifically, using exercise training and limb immobilization to alter capillarity, DO_2_ was completely dissociated from capillary density.

Myth 8: *Mitochondria in myocytes are distinct bean-shaped noninterconnected organelles*.

#### Evidence Falsifying Myth 8

**Figure 7.**
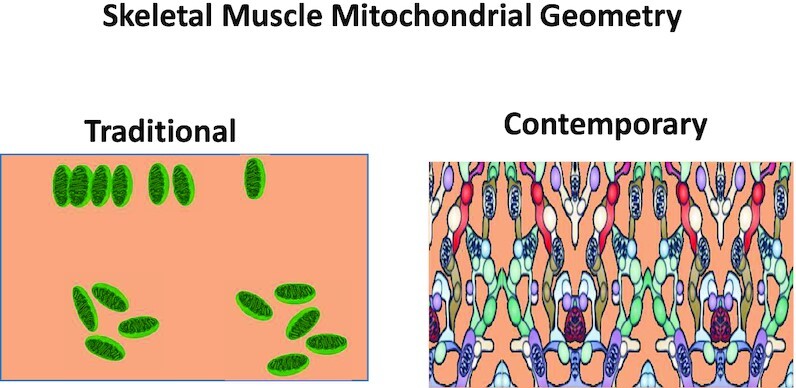
Over the past half-century it has become appreciated that mitochondria in skeletal muscles of humans and animals are not isolated bean-shaped organelles as depicted at left but, rather, form a catenated, interconnected reticulum that may transduce electrical charge (and potentially O_2_) across and along the myocyte (see right). This realization challenges traditional notions of O_2_ diffusion distances.

**Figure 8.**
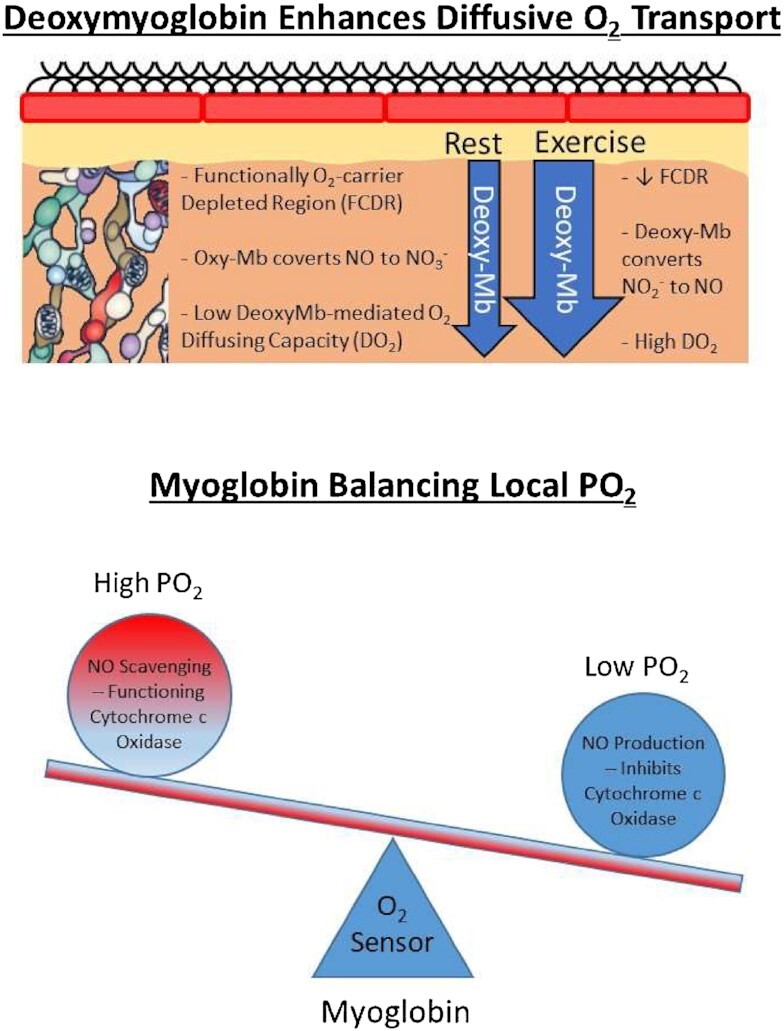
*Myoglobin-mediated Regulation of O_2_ Diffusing Capacity (DO_2_) and Oxidative Metabolism*. (Top) Mb molecules are saturated with O_2_ in resting muscle forming a functionally O_2_-carrier depleted region (FCDR) with low DO_2_. With exercise, Mb becomes deoxygenated as O_2_ utilization increases and ATP production accelerates; reducing the FCDR and elevating DO_2_. (Bottom) Mb molecules function as O_2_ sensors, spatially limiting, or facilitating cytochrome c oxidase activity via nitric oxide [NO] removal or production. Thus, locales with higher O_2_ partial pressure [PO_2_], will have NO scavenged by oxymyoglobin permitting uninhibited cytochrome c oxidase function whereas those with very low PO_2_ and deoxymyoglobin will reduce nitrite [NO_2_-] to NO, which will inhibit cyctochrome c oxidase activity and maintain PO_2_. This mechanism may be important for preventing formation of anoxic loci during maximal exercise (based upon^205^).

Viewed most commonly histologically in transverse muscle slices, intramyocyte mitochondria appear to be independent “bean-shaped” organelles reminiscent of their bacterial origins. In marked contrast, when viewed 3D intramyocyte mitochondria, in all principal fibre types, constitute an interconnected reticulum reaching from the subsarcolemmal space to the most capillary-distant intermyofibrillar mitochondria (see Figure 7).^197–203^ Moreover, acutely increased mitochondrial interconnectivity^204^ may potentially help explain the dramatically elevated intramyocyte O_2_ transport and DO_2_ found during exercise. That greater [cytochrome C oxidase complex IV] is found in subsarcolemmal versus intermyofibrillar mitochondria but [ATP synthase complex V] is not^202^^; rev.^^205^ might enable intramyocyte H^+^-electrochemical (and O_2_) gradient tracking. Much like a power grid, charge created at one location can power ATP production at a spatially remote site. Such a system would defend local ATP production in the face of very low PO_2_ and small PO_2_ gradients.^205^ In combination with oxy/deoxy-Mb removing/producing NO to regulate cytochrome C oxidase activity such an interconnected power grid would optimize ATP production in the face of a very low intramyocyte PO_2_ (Figure 8).

This schema would: (1) Abolish dependence on long intramyocyte capillary-mitochondria O_2_ diffusion pathways and creation of Krogh’s anoxic “lethal corners,” and (2) Negate the necessity for precise capillary positioning around the fibers.

### Role of Pericytes in Controlling Skeletal Muscle Capillary Hemodynamics

Reminiscent of Krogh’s search for a mechanism by which individual capillaries might control their own hemodynamics, it has been claimed that pericytes exert a “canonical” role in regulating skeletal muscle vessel diameter.^206^ However, some of the strongest evidence that pericytes can constrict/dilate capillaries comes from other tissues, most notably brain^207–210^ and heart muscle.^211,212^^; rev.^^213^ In particular, beautiful electrophysiological studies by Zhao and colleagues^211^ in mouse papillary muscle demonstrate that pericytes are electrically connected via gap junctions to ventricular myocytes. Thus, they propose that, when intramyocyte [ATP] ([ATP]*im*) falls, K_ATP_ channel opening hyperpolarizes myocytes, capillary endothelial cells, pericytes, and vascular smooth muscle cells; decreasing pericyte and vascular smooth muscle [Ca^2+^] and relaxing any extant constriction. This elegant potential mechanism would be exquisitely sensitive to Q̇O_2_-V̇O_2_ mismatch elevating Q̇O_2_ rapidly and specifically to ventricular myocytes with the greatest needs. That said, under physiological conditions, neither cardiac nor skeletal myocytes undergo any appreciable fall in [ATP]*im* even across substantial—orders of magnitude—increases of metabolic demand.^214,215^ Indeed, as demonstrated by Cain and Davies,^214^ it is necessary to completely inhibit creatine phosphoryltransferase with FDNB (1-fluoro-2,4-dinitrobenzene), a markedly nonphysiologic condition, to decrease [ATP] substantively during muscle contractions. Such a system might be better triggered, at least in skeletal muscle, by sensitivity to the phosphorylation potential (i.e., [ATP]/[ADP + Pi]).^216^

Moreover, even under highly nonphysiological conditions (e.g., capillary PO_2_ 140 to 700^+^mmHg) the extent of the pericyte-associated luminal constriction was extremely small (i.e., ∼20%, 1.2 µm;^210^ ∼13%,^211,212^) and thus, whereas it might increase individual capillary resistance, it would not be expected to prevent RBC passage. In response to whisker stimulation in mice, somatosensory cortex capillaries dilated ∼5% to 7% of their 4.4 µm baseline within ∼4 to 10 s^208^—faster than some arterioles and considered, by the authors, to be responsible for 84% of the regional blood flow increase. These kinetics are far faster than measured for bulk brain blood flow^217,218^ where the mean response time is ∼80 s suggesting that pericyte action may need to redistribute the existing blood flow in response to elevated regional demands. Interestingly, in brain, ischemia induces pericyte constriction of capillaries which may be irreversible.^208^

Capillary perfusion heterogeneity may not be controlled in muscle ^219^ as it potentially is in brain.^125,220,221^ It is also pertinent that, in heart, pericyte coverage is less than in brain with not all capillaries having a pericyte.^211,212^ Specifically, endothelial cells are ∼10 × 30 µm in size^222^, whereas brain and heart have an endothelial cell-to-pericyte ratio between 1:1 and 1:3^223,224^ in skeletal muscle it is 100:1^225^^; rev.^^226^ decreasing the opportunity for many capillaries to even have a pericyte. Also, whereas some authors claim that pericytes play a deterministic role in muscle perfusion, the strongest evidence for such, at present, is restricted to pathological states such as arterial stenosis.^227^ It is also pertinent that, of the three tissues considered above, skeletal muscle has by far the greatest range of metabolic rates demanding over a 100-fold increase in blood flow from rest to maximal exercise.^126^ Thus, the potential for, but perhaps not the consequences of, Q̇O_2_-V̇O_2_ mismatch is far greater in skeletal muscle versus heart or brain.

In conclusion, for pericyte relaxation to account for the rapid (∼1 s or less) skeletal muscle capillary hyperemia following the onset of contractions (see *Myth #6* above) it would be necessary to demonstrate several phenomena; including: (1) That, in resting muscle, pericytes are constricting capillaries and occluding RBC flux. As evidence falsifying *Myth #1* above (see also Table 1, right column) substantiates, this does not appear to be the case. (2) That pericytes can relax almost simultaneously across most of the capillary bed in synchrony with the first contraction–relaxation cycle following the onset of exercise.

Perhaps, there might be a role for pericytes improving Q̇O_2_-V̇O_2_ matching in the secondary phase of the muscle capillary hyperemic response wherein P*mv*O_2_ falls, and a-vO_2_ difference rises, toward steady-state values.^89^ To date, however, direct evidence is lacking. Vasoaction, at least in response to exercise (onset and offset,^89,178^), chemical stimuli,^228,229^ and the K_ATP_-channel blocker glibenclamide,^101^ as well as the pathological derangements (decreased proportion of capillaries supporting RBC flux, impaired capillary hemodynamics, in HF,^90^ Type II diabetes,^92^) appear to be mediated exclusively at the arteriolar level. Specifically, capillary hemodynamics are accelerated in hyperemic states and decelerated in low flow conditions without any discernible narrowing of the capillary lumens whether caused by pericytes or other means.^229^ It is also pertinent that, in resting or contracting muscle at sarcomere lengths below ∼2.7 µm (i.e., physiological, nonstretched), capillary RBC velocity and flux through individual capillaries exhibit enormous heterogeneity and do not correlate with capillary luminal diameter.^85–89^, ^230^

### Oxygen Flux At the Blood-Myocyte Boundary: O_2_ Diffusing Capacity (DO_2_)

**Figure 9.**
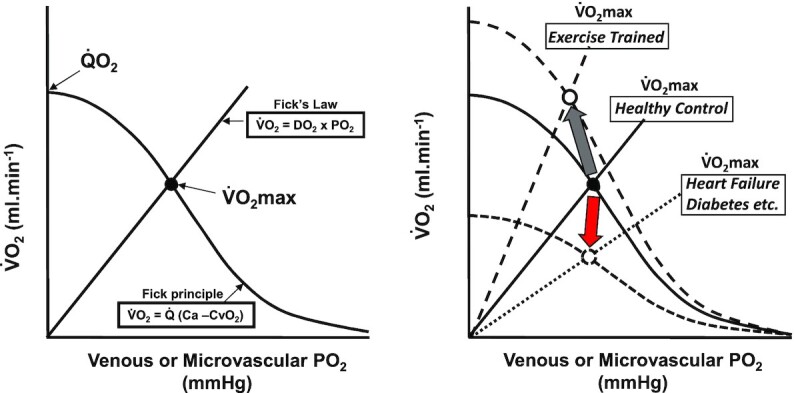
*Perfusive and Diffusive Oxygen Transport Determine Maximal Oxygen Uptake (V̇O_2_max)*. (Left) Perfusive [curved line, Fick principle: V̇O_2_ = muscle blood flow (Q̇) × fractional oxygen extraction (Ca-CvO_2_)] and diffusive O_2_ [straight line, Fick’s Law: V̇O_2_ = diffusing capacity for oxygen (DO_2_) × O_2_ partial pressure (PO_2_)] conductances conflate to determine V̇O_2_max. (Right) Resolving this relationship in health facilitates assessment of the mechanistic bases for plasticity invoked by exercise training (gray arrow, i.e., increased bulk blood flow and red blood cell distribution, DO_2_, ∆ PO_2_, etc.) and/or diseases such as heart failure or diabetes (red arrow, i.e., ∆ blood flow distribution, ↓capillaries flowing, ∆O_2_ extraction, ↓DO_2_, ∆PO_2_), and aging.^107,123,250^

For many years it was considered that acute and chronic (e.g., exercise training, aging, disease) changes in V̇O_2_max were driven almost exclusively by changes in muscle(s) perfusive O_2_ flux (i.e., Q̇ × arterial [O_2_] or Q̇O_2_) with little attention paid to muscle O_2_ diffusing capacity (DO_2_). However, some 3 decades ago Peter D. Wagner graphically (Figure 9) conflated the Fick principle


(1) $$\begin{equation*}
\dot{V}{O}_2 = \dot{Q}\left( {Ca{O}_2- Cv{O}_2} \right),
\end{equation*}$$

(2) $$\begin{equation*}
{\rm{with\,Fick \rm^{\prime}s\,Law\!:\dot{V}}}{{\rm{O}}}_{\rm{2}}{\rm{ = D }}\left( {{\rm{Pcap - Pmito}}{{\rm{O}}}_{\rm{2}}} \right),
\end{equation*}$$

which demonstrated effectively that the reason skeletal muscle could not remove all the O_2_ from the venous effluent, at V̇O_2_max when the mitochondrial were O_2_-supply limited, was the presence of a finite DO_2_.^231,232^^; rev.^^233^ As demonstrated in Figure 9, right panel, measured venous (or calculated mean capillary) PO_2_ or O_2_ content may be little impacted by exercise training or HF, for example, but conceal substantial changes in DO_2_. For instance, despite only a small increase in a-vO_2_ difference, in response to exercise training ∼70% of the increased V̇O_2_max is attributed to elevated DO_2_ rather than Q̇O_2._^232^ For HF patients a reduced DO_2_ contributes substantially to their lowered V̇O_2_max even though muscle venous effluent PO_2_ may be very low.^234^^; rev.^^195^ Thus, fractional O_2_ extraction is synonymous with a-vO_2_ difference and determined by the ratio of DO_2_-to-Q̇


(3) $$\begin{equation*}
{\rm{Fractional\, }} {{\rm{O}}}_{\rm{2}}\, {\rm{extraction = \dot{V}}}{{\rm{O}}}_{\rm{2}}{\rm{/\dot{Q}}}{{\rm{O}}}_{\rm{2}}{\rm{ = \dot{Q}}}{{\rm{O}}}_{\rm{2}}\left( {{\rm{1 - }}{{\rm{e}}}^{{\rm{ - DO2/\beta \dot{Q}}}}} \right),
\end{equation*}$$

where β is the slope of the O_2_ dissociation curve in the relevant physiological range.

That the number of RBCs immediately adjacent to the muscle fibers within flowing capillaries is a primary determinant of DO_2_^108^,^109^,^193^ coheres with the capillary neogenesis stimulated by exercise training increasing, ^rev.^^235^,^236^ and the capillary involution and high proportion of capillaries that do not support RBC flux in HF decreasing, ^90,194^ DO_2_ (Figure 9, right panel).

**Figure 10.**
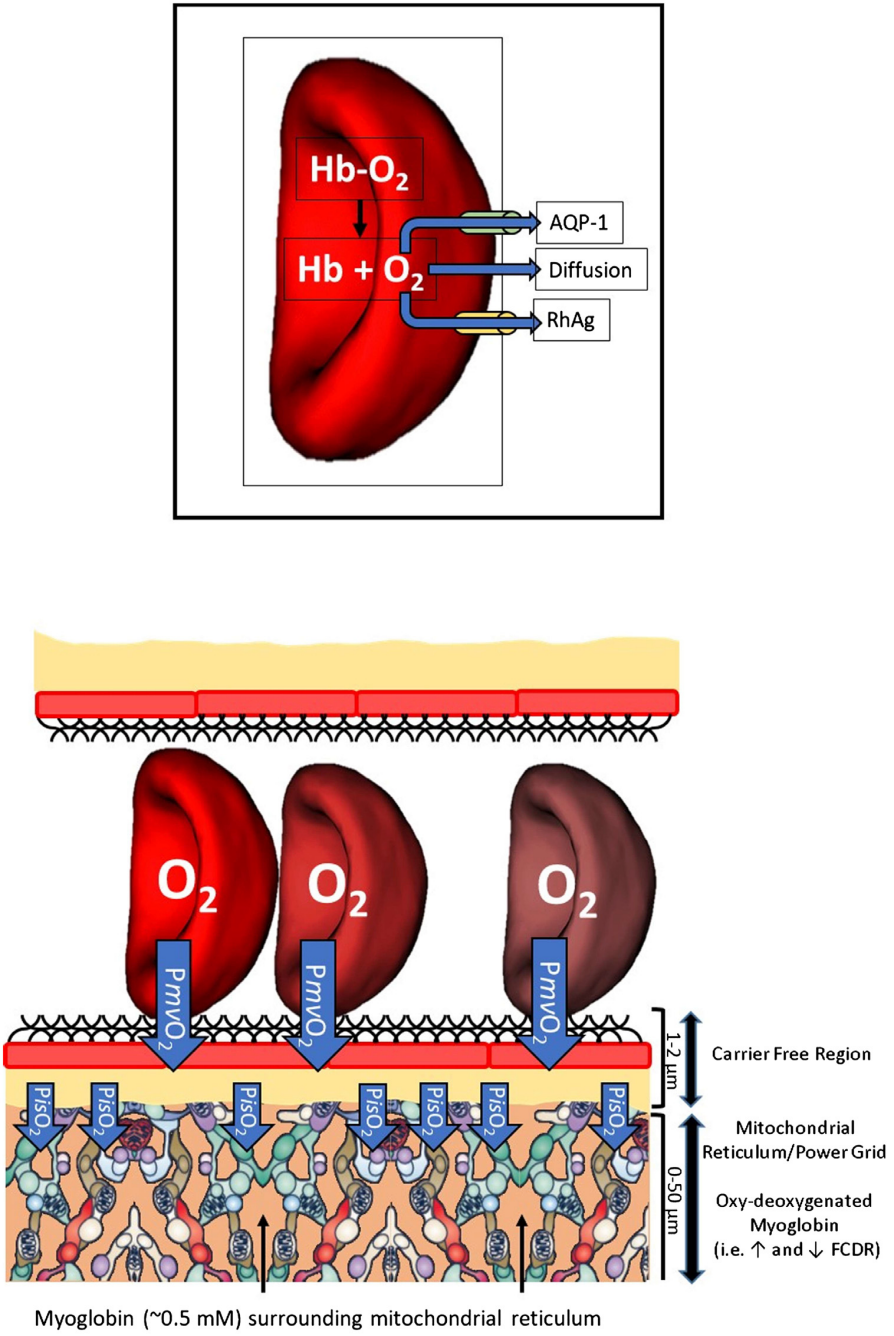
Key Features of Red Blood Cell-to-Intramyocyte Oxygen Transport. Muscle O_2_ uptake (V̇O_2_) increases rapidly and exponentially following the onset of exercise. Haemoglobin-bound O_2_ in red blood cells (RBCs) moves down its pressure gradient at the RBC-capillary interface (P*mv*O_2_, microvascular partial pressure for O_2_) crossing plasma, the endothelial surface layer/glycocalyx, endothelial cells, and on into the interstitial space. In addition to simple diffusion, movement of O_2_ across the RBC plasma membrane occurs via aquaporin (AQP-1) and rhesus (RhAg) channels.^241,242^ O_2_ carriers being absent between the RBC and sarcolemma (carrier free region), differences in functional surface area available for O_2_ flux produce a higher RBC-interstitial O_2_ flux density than that at the interstitial-intramyocyte barrier: Requiring a substantial pressure differential (i.e., ∆[P*mv*O_2_ > P*is*O_2_], interstitial space PO_2_) to transcend the apparent O_2_ flux resistance between compartments. As seen in Figure 7 subsarcolemmal interconnect with interfibrillar mitochondria^202,203^ and are surrounded by cytoplasmic Mb. When O_2_ saturated in muscle at rest, oxymyoglobin forms a functionally depleted O_2_-carrier region (FCDR) that desaturates during exercise increasing O_2_ diffusing capacity and O_2_ transport to the more “distant” mitochondrial reticulum (see text and Figure 8 for further details, rev.^135^).

As O_2_ moves down its pressure gradient from capillary RBCs to mitochondria in skeletal muscle to power oxidative energetics it must (in sequence): Dissociate from hemoglobin, traverse the RBC membrane, plasma, capillary endothelial surface layer (glycocalyx), endothelial cell, interstitial space, myocyte sarcolemma and intervening cytoplasm, cross the mitochondrial membranes, and react with reduced cytochrome c oxidase and protons (Figure 10). ^rev.^^107^ Whereas each of these steps will provide some finite resistance, measurements of P*mv*O_2_ and interstitial PO_2_ (P*is*O_2_) by phosphorescence quenching and intramyocyte PO_2_ (P*im*O_2_) by proton NMR and cryomicrospectrophotometry support that the bulk of the apparent resistance to O_2_ flux lies in close proximity to the capillary. Thus the majority of the PO_2_ decrease between RBC and mitochondria occurs from capillary to interstitial space and from there into the myocyte (i.e., ∼1 µm).^131–133^ During contractions the P*im*O_2_ is low (i.e., 1 to 5 mmHg in human vastus lateralis)^140^ and absent of marked transverse or longitudinal gradients (canine gracilis).^135^

Let’s consider individual steps in O_2_’s pathway from RBC to mitochondria.

#### Red Blood Cell

Hemoglobin O_2_ dissociation has a 4 ms half-time with a velocity constant ∼3 000 mM/s under physiological conditions.^237–239^ At V̇O_2_max, PO_2_ decreases <4 mmHg from Hb to the RBC membrane.^240^ Exciting recent data suggest that two channels (aquaporin-1, AQP-1, rhesus, RhAg) facilitate transmembrane O_2_ flux^241^ and may account for over half of the RBC O_2_ diffusing capacity.^241,242^ It remains to be determined how knocking out these channels impairs skeletal muscle DO_2_ and exercise capacity in vivo.

#### Plasma, Glycolcalyx, Endothelial Cell, And Interstitial Space

Oxygen’s insolubility in plasma dictates that only RBCs themselves are quantitatively important O_2_ sources. Thus the proportion of the capillary endothelium relevant for O_2_ flux is determined by how many capillaries sustain RBC flux as well as their length and *crucially* their hematocrit.^108,109^ Acting like an O_2_ bottleneck the RBC-endothelial region concentrates the O_2_ flux density forcing a large fall in PO_2_ across the microvascular-to-interstitial space.^131–133^ This bottleneck is widened during exercise as capillary hematocrit increases from 10% to 20% at rest toward systemic values (∼45%).^rev.^^119^ It remains to be determined exactly how high capillary hematocrit rises during heavy or severe intensity exercise. Measurements using Time Resolved Near Infrared Spectroscopy (TRS-NIRS) in human muscles during severe intensity exercise indicate that muscle hematocrit may only increase up to 30% above resting^121,122^,^243–245^ contributing only fractionally to the DO_2_ elevation necessary for the 100-fold increase in blood-muscle O_2_ flux.

With respect to P*mv*O_2_ and P*is*O_2_ there is a marked fibre type heterogeneity. Specifically, Q̇O_2_ at rest and during exercise is maintained far higher, with respect to V̇O_2_ (i.e., ↑ Q̇O_2_-to-V̇O_2_ ratio) for slow- versus fast-twitch muscles, such that their P*mv*O_2_ is higher.^133,138,139^ Quantifying the PO_2_ profile across the microvascular and interstitial compartments at rest and during contractions within contrasting fibre-type muscles helps partition the apparent O_2_ flux resistance into myocytes. The 1-2 µm region from the RBC membrane to the myocyte subsarcolemmal space is considered an O_2_ carrier-free region (CFR) encompassing the plasma and endothelial surface layers, endothelial cell, interstitial space and sarcolemma (Figure 10). Interestingly, the substantial trans-capillary PO_2_ gradient is similar in both slow- and fast-twitch muscles at rest and during contractions (1 Hz, ∼moderate intensity exercise). Thus, despite the contractions-induced increase of capillary hematocrit, the plasma-glycocalyx-endothelial cell interface presents an appreciable apparent resistance to blood-myocyte O_2_ flux amounting to ∼60% (soleus) to 85% (white gastrocnemius) of the capillary-myocyte PO_2_ drop: which is consistent with Roy and Popel’s^246^ theoretical modeling in athletic and nonathletic animal muscles.

In slow- versus fast-twitch muscles the higher P*mv*O_2_ (rest and contracting) and greater P*is*O_2_ promotes a higher trans-sarcolemmal O_2_ flux and may serve to sustain a higher intramyocyte PO_2_ (P*im*O_2_). This higher P*im*O_2_ will reduce metabolic perturbations (i.e., less increase of [ADP]_free_, [Pi], [H^+^], and glycolysis) and improve muscle contractile performance.^141^,^247–249^ That upstream vascular regulation can dictate muscle metabolic control across fiber types has been heretofore marginalized by attention almost solely to fiber enzymatic properties. Greater focus on this aspect of vascular control may provide novel insights into exercise intolerance in health and especially major diseases such as HF and diabetes.^rev.^^1,2^ ^123^, ^124^,^195^,^250^

### Intramyocyte O_2_ Transport: Sarcolemma-Mitochondria

The theoretical model of Krogh and Erlang hypothesized that muscle PO_2_ decreased proportionally with distance “R” from the capillary (Figure 2 #4).^21–23^ The foundations for that model included: Muscles have a finite “diffusivity” that is increased from rest-exercise solely by recruitment of more capillaries each with a unitary perfusive and diffusive capacity supplying a fixed volume of tissue that increases with distance from the capillary (see *Myth #4* above). Muscle regions distant from capillaries exist in an anoxic state (PO_2_ = 0 mmHg^see also^^251^) at rest and become oxygenated during contractions. The role of Mb in intramyocyte O_2_ transport was not appreciated.

Decades later development of the PO_2_ microelectrode facilitated construction of tissue PO_2_ histograms revealing mean PO_2_ values between 16 and 39 mmHg in resting skeletal muscles.^252^^; rev.^^253^ Later Honig and colleagues’^135^ cryomicrospectrophotometry resolved submyocyte myoglobin O_2_ saturations, which permitted calculation of local intramyocyte PO_2_’s in rapidly frozen resting and contracting dog gracilis muscles. Despite technical shortcomings, that precluded spatial resolution much below 50 µm,^254^ their data showing low intramyocyte P*im*O_2_s during contractions—and therefore absence of appreciable intramyocyte PO_2_ gradients—was confirmed by proton-NMRS in human muscles. Specifically, in human muscles P*im*O_2_ was reduced from 20 to 30 mmHg at rest to between 2 and 5 mmHg during heavy-maximal exercise (Figure 3 #4).^140–143^,^255^

Within human muscles in particular there is an interdigitating mosaic of contrasting fibre types and a dissociation anatomically between capillary modules and muscle fibers within an individual motor unit. Accordingly, the same capillaries may supply both quiescent and active muscle fibres^256^^; rev.^^102^ contributing to disparate Q̇O_2_-to-V̇O_2_ and thus variant extraction ratios across fiber types^16^ and microregions. Given this behavior it is remarkable that the femoral arterial–venous O_2_ difference for human cycling exercise increases (and femoral venous O_2_ concentration decreases) hyperbolically^1^ according to:


(4) $$\begin{equation*}
{\rm{Ca}}{{\rm{O}}}_{\rm{2}}{\rm{ - Cv}}{{\rm{O}}}_{\rm{2}}\left( {{\rm{ml/100 ml}}} \right){\rm{ = 22\dot{V}}}{{\rm{O}}}_{\rm{2}}{\rm{/}}\left( {{\rm{1 + \dot{V}}}{{\rm{O}}}_{\rm{2}}} \right),
\end{equation*}$$

where V̇O_2_ is in l/min.

Any model of capillary function and O_2_ movement from capillary-mitochondria must be consistent with the physiological observations characterized in equation 4.^16,119,125^ It is argued below that a contemporary understanding of O_2_ movement across/within myocytes is not consistent with the necessity for de novo capillary recruitment from rest-exercise nor the importance of capillary–mitochondrial diffusion distances with respect to O_2_ supply.

#### Modeling Capillary-Myocyte O_2_ Delivery

Abundant evidence demonstrates that, in skeletal muscles, the locus of capillary RBC flux control resides in the arterioles with some modulation from vasodilation signals arising from within individual capillaries.^257–262^ These signals are conducted via gap junctions^rev.^^259^ and also a pannexin/purinergic-dependent pathway^262^ and contribute to the rapid hyperemia (i.e., <1 s).^89,183,259^ There may also be fine-tuning of capillary RBC flux across capillary modules resulting from venular vasomotor control.^102^ As discussed above, (see the section Role of Pericytes in Controlling Skeletal Muscle Capillary Hemodynamics) evidence for unitary capillary RBC flux modulation by pericytes (Rouget cells) in skeletal muscle—at least in health and with kinetics that can account for the increased capillary RBC flux measured—is wholly absent. Moreover, the presence/participation of precapillary sphincters^21–23^,^25,50^^; rev.^^158^ or a contractile endothelium are not supported by either anatomical or physiological evidence.

In *Myth #3*, previously, we considered that the capillary 3D geometry, tortuosity, and branching, particularly in passively shortened or contracting muscles,^128^ supports the Hill model of O_2_ delivery. Critically, for the Hill model the dependent tissue volume *decreases* with distance from a given capillary and/or the myocyte sarcolemma (Figure 5, right panel)^130^ with all capillaries adjacent to a particular fibre cooperatively supplying O_2_ in proportion to their convective and diffusive O_2_ conductances and the fibre’s metabolic demands.^89^^; rev.^^107,125^ It is also notable that capillaries may also target their O_2_ delivery preferentially to 1 of 2 or 3 adjacent fibres by selectively embedding into the sarcolemma of that fibre.^263^

#### O_2_ Interdependence of Microvessels and Myocytes

Countercurrent capillary flow is present, but it not considered to contribute much to arteriovenous O_2_ shunting.^86,264^^; rev.^^253^ However, capillaries with short RBC transit times, consequent either to minimal path lengths^265^ or high RBC velocities, can potentially decrease fractional O_2_ extraction.^253^ That said, in human knee extensor muscles, it is possible to achieve ∼80% O_2_ extraction at estimated mean capillary RBC transit times of only 0.1-0.2 s at Q̇’s ∼4 l/kg/min. ^126,127^ This process may be aided by the close proximity of arterioles, tortuous capillaries and venules and their anastomoses facilitating diffusive interactions among adjacent capillaries, capillaries and arterioles as well as venules (Figure 4 #5): ^136,137,266^ A process where the interstitial space must constitute a conduit for O_2_ around the periphery of the myocytes (Figure 5, right panel). These “network” O_2_ diffusional interactions are likely to be crucial to support the rapid V̇O_2_ kinetics following exercise onset where, in young healthy fit individuals, Q̇O_2_ kinetics being at least as fast as mitochondrial V̇O_2_ kinetics such that mitochondrial energetics, rather than Q̇O_2_, constrain V̇O_2_ kinetics.^1,2^

#### Traditional Role of Mb

Mb has been regarded as an O_2_ reservoir and, particularly in red, highly oxidative muscles, a primary O_2_ transporting molecule^267–269^^; rev.^^270^ with both functions determined by [Mb] and extent of desaturation during exercise. In keeping with an important role for Mb in intracellular O_2_ transport Mb-O_2_ conductance correlates with muscle oxidative capacity across species^271^^; rev.^^272^ and [Mb] with animal mass across several orders of magnitude body mass from pigeon to blue whale (i.e., psoas muscle data from^273^^; rev.^^272^). In addition, for horses and steers running at V̇O_2_max, muscles with the highest [Mb] were those calculated to have the greatest diffusion limitation (i.e., V̇O_2_/DO_2_ > 1).^272^ An extensive modeling literature also supports the importance of Mb-facilitated intramuscular O_2_ transport.^274^ ^275^^; rev.^^276^

##### Mb As an O_2_ Store

Diving mammals may have extremely high [Mb] in their muscles^273^^; rev.^^272^ but for terrestrial mammals and especially human locomotory muscles [Mb] may only be ∼0.5 mM.^277^^; rev.^^276^ Thus, in the extreme case of maximal exercise in human muscle (P*im*O_2_ 2-3 mmHg giving ∼50% Mb desaturation) ^140^ this ∼6 ml O_2_/kg represents just <1 s of the extant V̇O_2_.

##### Mb As a Facilitator of Intramuscular O_2_ Diffusion

Although championed by the Wittenbergs^278–280^^; rev.^^266^^; see also^^253^ Mb’s function as an O_2_ transporter is confounded by its large mass of 17,500 kDa and 3.5 nm diameter, which restricts mobility and compromises diffusion.^280^^; rev.^^107,266^ Considering its size and concentration the effective conductance of Mb-O_2_ is so low that it is thought to approximate that of free O_2_.^272,276^ Accordingly, whether Mb has a quantitatively important role in intramuscular O_2_ transport has been questioned.^281^ ^282^^; see also^^ref. 283^^for low Mb diffusivity^ That said, during exercise Mb does partially O_2_ desaturate in contracting muscles (humans,^255^,^140–143^; canine,^135^) permitting development of an Mb-O_2_ gradient facilitating Mb-mediated O_2_ diffusion. Thus, at low metabolic rates/rest P*im*O_2_’s ∼20 to 30 mmHg^142,143^ essentially fully oxygenate Mb and would therefore extend the CFR (O_2_ carrier-free region) from sarcolemma into the myocyte precipitating a so-called functionally O_2_-carrier depleted region (FCDR).^125^ With Mb deoxygenating during exercise greater O_2_ transport capacity would be recruited as the FCDR disappears thereby increasing muscle DO_2_. Although speculative, this Mb mechanism (reduced FCDR) may potentially explain a portion of the rest-exercise increase of DO_2_ that *cannot* be attributed to events in the capillary (i.e., elevated hematocrit and increased longitudinal recruitment of endothelial surface area).^51,119,124,125^

The fact that Mb-less mice can exercise^284^ would seem to mitigate against an obligatory role for Mb. However, these animals do exhibit high *in utero* mortality, greater systemic hematocrit and coronary blood flow reserve and capillarity but impaired cardiac and exercise performance.^285,286^ It is not presently known whether larger species of Mb knock-outs with greater muscle fibre cross-sectional areas are viable.

Notwithstanding the above support for Mb’s importance in facilitating intramyocyte O_2_ transport there is evidence that diffusion distances, in-and-of themselves, might not limit O_2_ delivery. If this were indeed the case one *raison d’etre* for Mb would not exist. Specifically, Hudlicka and colleagues^287^ noted that muscles with contrasting intercapillary distances exhibited very similar muscle/mitochondria-specific metabolic rates.^see also^^288^ ^289^ Also, DO_2_ at V̇O_2_max in dog gastrocnemius-plantaris is not related to capillary density and therefore mean intramuscular diffusion distances after immobilization or exercise training.^196^ Unfortunately, those studies could not determine the RBC volumes within the capillary bed, and changes thereof, that constitute a primary determinant of DO_2_.^108,109^

In addition to this circumstantial evidence questioning the central role for Mb-facilitated O_2_ diffusion (and appreciable O_2_ storage in terrestrial mammalian muscles) Gros et al.^276^ have calculated that, given the root mean square displacement for Mb-O_2_ between ∼4 to 12 µm, O_2_ must combine and dissociate multiple times to traverse the necessary intramyocyte distances (e.g., up to 10 to 60 µm). Moreover, Mb-O_2_ must cross various potential barriers such as sarcoplasmic reticulum, myofilaments and T-Tubules although M-Lines and Z-disks may not impede this process.^282,290^^; rev.^^107^ It is also pertinent that, whilst the low intramyocyte PO_2_’s^135,140,143^ might facilitate O_2_ transport via Mb, in part by removal of the FCDR, absence of substantial intramyocyte PO_2_ gradients, from subsarcolemmal to more capillary-distant cell regions, would presumably decrease the quantitative importance of passive relative to Mb-facilitated O_2_ diffusion.

#### Contemporary Role for Mb—The Intramyocyte Power Grid

Both cryomicrospectrophotometric^135,254^,^291–293^ and P-NMR^140,142^ analyses, though spatially crude, support that intramyocyte PO_2_’s remain >1 mmHg. This value is far greater than the “critical” PO_2_, below which V̇O_2_ is constrained (i.e., 0.1 to 0.5 mmHg).^294^ In contrast, mitochondrial V̇O_2_max versus intramyocyte PO_2_ curves generated by humans inspiring a range of hypoxic, normoxic, and hyperoxic gases (i.e., seeFigure 8 in^272^) support a far higher critical PO_2_ ∼4 mmHg; as do measurements made by phosphorescence quenching in the rat spinotrapezius muscle.^295,296^ Interestingly, spectroscopic determination of muscle [oxidized cytochrome C oxidase (a, a_3_)] at V̇O_2_max reached a nadir common with that for death or anoxia,^297^ which were interpreted as evidence for an ∼2 mmHg cytosol-mitochondrial PO_2_ gradient.^272^ However, with an estimated mitochondrial surface area being >100 times that of the functional capillary (i.e., adjacent to flowing RBCs) the corresponding O_2_ flux density would be tiny; requiring a disappearingly small transmitochondrial membrane(s) PO_2_ gradient.^240,298,299^

##### Mb As an O_2_ Sensor and Signaling Molecule

Beyond any storage and/or O_2_ transport function of Mb the novel concept has been advanced that, depending upon local O_2_ availability, Mb switches between NO scavenging and NO production. Thus, at higher PO_2_’s, extant for example in resting muscle, Mb continuously produces nitrate (NO_3_^−^) from NO raising intramyocyte [NO_3_^−^] above plasma concentrations (rodents^300^; humans^301^). When intramyocye PO_2_ falls precipitously during intense exercise (Figures 3 #4 and 4 #7)^rev.^^205^ Mb, located perimitochondrially, produces NO via xanthine oxidoreductase reduction of NO_3_^−^ to NO_2_,^−^ deoxy-Mb then reacts with NO_2_^−^ to form deoxymetMbNO(Fe^+3^) from which free NO dissociates, triggering NO-mediated suppression of oxidative function.^302–304^ This process, potentiated by the elevated [H^+^], [xanthine], and [hypoxanthine]^305^ transposes the energetic burden from intermyofibrillar to subsarcolemmal mitochondria thereby preserving intramyocyte O_2_ flux and forming what Clanton^205^ has insightfully termed the “NO shield” (see Figure 8). Although, in many circumstances, elevating [NO] and increasing its bioavailability is an important clinical goal, there is evidence that too much intracellular NO production, for instance by iNOS in response to sepsis, reduces oxidative function.^306^ That increased iNOS-generated NO in sepsis, and other diseases, might impair muscle energetics and contractile function by compromising the NO shield’s ability to distribute oxidative function across the mitochondrial reticulum, is an intriguing mechanistic hypothesis.

#### Skeletal Muscle Mitochondria: Structure-Function and Relevance for Understanding Capillary-Mitochondrial O_2_ Transport

In contrast to the classical depiction of mitochondria as independent “bean-shaped” organelles, in myocytes, of all mammalian fibre types, they constitute a contiguous interconnected reticulum (see Figure 7) that may extend from subsarcolemma to intermyofibrillar regions.^197–203^ Moreover, the structure of the mitochondrial reticulum itself is not fixed but dynamically modulates, potentially becoming more interconnected during exercise.^204^ This observation, if confirmed, may help explain how exercise promotes such large increases in intramyocyte O_2_ transport and DO_2_. In addition, the composition of the mitochondrial reticulum is heterogeneous with respect to subsarcolemmal mitochondria exhibiting greater [cytochrome C oxidase complex IV] than their intermyofibrillar whereas [ATP synthase complex V] is homogeneous.^202^^; rev.^^205^ These observations underpin the capacity for H^+^-electrochemical (and likely lipid/free fatty acids and O_2_) gradients to move spatially like a power grid. In this manner charge generated at one location can produce ATP remotely. The elegance of this system provides a robust protection against focal hypoxia limiting ATP generation and abrogates dependence on long O_2_ diffusion pathways from capillary to mitochondria. Without the opportunities for the “lethal corners” of tissue anoxia hypothesized by Krogh, this schema helps explain the dissociation between capillary density (therefore diffusion distances) and DO_2_ noted by Hepple and colleagues.^196^^; see also^^287–289^

Among species and muscle fibre types and across exercise training programs muscle DO_2_ can correlate with capillary volume density.^232^,^307^ As above, this observation is consistent with DO_2_ being determined principally by the volume of RBCs adjacent to the contracting muscle fibers^108,109^^; see also^^193^ and mandates using 3D models with RBC volume fractions to calculate muscle oxygenation.^308^ The DO_2_ dependency on capillary RBC volume means that RBC distribution and hemodynamics can, via altered DO_2_, impact P*im*O_2_ regulating muscle metabolic control and therefore contractile performance.^141^, ^247–249^

**Figure 11.**
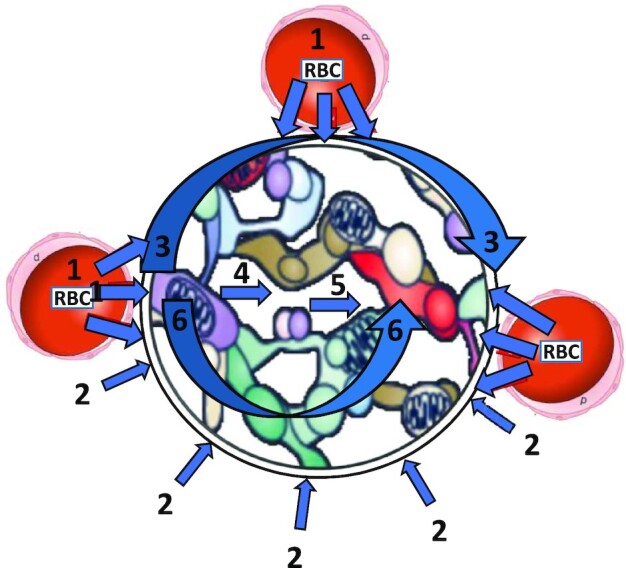
Mechanisms for Red Blood Cell (RBC)-Mitochondria O_2_ Transport in Skeletal Muscle. (1) O_2_ (blue arrows) moves across the RBC membrane through aquaporin and rhesus channels, down its pressure gradient across the plasma and endothelial cells into the interstitial space. (2) Interstitial O_2_ transport. (3) O_2_ tracks along sarcolemmal phospholipid bilayer. (4) Simple diffusion through cytosol. (5) Myoglobin-facilitated diffusion. (6) H^+^-electrochemical gradient (and perhaps O_2_) track across interconnected mitochondrial reticulum. See text for more details.

In addition to upstream (i.e., extramyocyte) determinants of DO_2_ it is important to consider all the potential pathways of intramyocyte O_2_ transport (Figure 11). We have, based upon capillary 3D geometry, made the case for the Hill model of O_2_ diffusion being more appropriate than Krogh’s “pin-point” capillary O_2_ supply. In addition, the demonstrated movement of O_2_ among adjacent capillaries and other vessels supports that the interstitial space, which has a greater PO_2_ than P*im*O_2_,^131–133^ constitutes an alternative/additional pathway for trans-sarcolemmal O_2_ flux. Such a mechanism could greatly increase the available surface area, which now becomes the entire sarcolemma, for O*_2_* diffusion (and hence DO_2_) above that provided by the direct capillary–sarcolemmal “contact” considered by Ellis and colleagues when proposing the Hill model.^130^

Thus, the geometry of intramyocyte O_2_ transport is very different from that hypothesized in the Krogh model. Tissue and mitochondrial volume decrease with increasing distance from the sarcolemma (c.f., Figure 5, left and right) with O_2_ moving around and into the myocyte at sites remote from capillaries. Intramyocyte PO_2_ profiles are flat with PO_2_ gradients not detectable. Within the myocyte there will be free O_2_ and Mb-facilitated O_2_ transport—though the absolute and relative importance of each is debated especially given the absence of substantive O_2_ gradients.^276^ In addition, Pias^309^ has presented an intriguing, though mostly theoretical, case for O_2_ transport tracking along high O_2_-conductance lipid membranes. Thus, there are at least 3 putative parallel paths for intramyocyte O_2_ transport (Figure 11). These pathways, conflated with the ability of the mitochondrial reticulum to track H^+^-electrochemical gradients deep into the fiber,^197–203^ would, presumably, mitigate (or remove entirely) O_2_ flux constraints resulting from capillary-mitochondrial O_2_ diffusion distances.

### Intravital Microscopy of Skeletal Muscle: Essential Quality Control

In contrast to the right side of Table 1 (opposing capillary recruitment) the left side exemplars studies in which it is concluded that most (or many) capillaries in resting skeletal muscle do not support RBC flow at rest and are thus available to be recruited during exercise. Whereas many papers interpret their indirect findings in terms of capillary recruitment without actually observing the capillary bed—a practice harshly criticized by the eminent microscopist Professor Eugene Renkin^310^—there are also intravital microscopy studies supporting that the majority of (or at least many) capillaries in resting muscle do not support RBC flow (see left side of Table 1). In this regard it is pertinent that capillaries are extremely delicate structures that can easily be damaged by the surgical intervention necessary to visualize the microcirculation and also the structure and function of which is impacted by muscle length (especially stretching), arteriolar control (neural and humoral), and their physicochemical microenvironment. The following are just a few of the experimental conditions, lack of attention to which, will decrease the proportion of capillaries supporting RBC flux and, as experienced by Krogh’s stretched, hyperoxic frog tongue preparation, set the stage for contraction-induced capillary recruitment.

1. Anesthesia, especially in combination with systemic hypoxia, can cause hypotension decreasing muscle Q̇ (and P*mv*O_2_) via reduced driving pressure and also a sympathetically induced arteriolar vasoconstriction.^311^
2. Local muscle hyperoxic and hypoxic conditions should be specifically avoided as they will produce nonphysiological vasoconstriction (hyperoxia) and muscle dilation (hypoxia). The former will have direct consequences for decreasing the proportion of capillaries that support RBC flux. Phosphorescence quenching measurements have defined the microvascular and interstitial PO_2_’s (i.e., P*mv*O_2_, P*is*O_2_) in resting and exercising muscles across the fibre-type spectrum (see Figure 3 #4)^131–133^ and it is now possible to set the appropriate physiological conditions, at least for PO_2_.
3. Sharp or blunt force trauma during surgery can prevent arterioles and capillaries flowing. Aggressive removal of overlying fascia to improve visual clarity may also damage the microcirculation and should be avoided/minimal.
4. Muscle stretch above 3 µm, often practiced to thin the muscle and improve microcirculation optical clarity, stretches the capillaries, reducing their luminal diameter and vascular conductance and stopping RBC flux in a substantial proportion of capillaries.^88^ Stretching muscles also produces a reflex vasoconstriction, which compounds the hyporemia and capillary no-flow.^312^

Attention to these and other physiological conditions (temperature, hormonal, fluid status) insofar as possible, is simply good science. Where such a standard is not met, the results must be interpreted accordingly. With respect to muscle oxygenation this is especially true, not just for microcirculatory, but also for metabolic control. Specifically, even disappearingly small changes in intramyocyte PO_2_ (P*im*O_2_), evoked consequent to altered P*mv*O_2_ and P*is*O_2_ (and thus P*im*O_2_) can exert a commanding influence on myocyte energetics, substrate utilization and metabolic control.^247–249^^; rev.^^313^^; see also^^141,314^ Moreover, inattention to establishing physiologic PO_2_ profiles can destroy our ability to, for example, determine the effect of manipulating the nitrate-nitrite-NO pathway on contractile performance.^315^

## Conclusions

Effective progress in muscle microcirculatory research will be facilitated by:

Advocating for rigorous quality control of intravital microscopy investigations and interpreting previous findings, theories, and models accordingly.

Recognizing that different organ microcirculations subserve wide-ranging functions and those elements critical for capillary hemodynamic control in muscle may work very differently for heart, mesentery, gut, and especially the brain.

Testing novel or extant hypotheses within the context of physiological control. For instance, as regards the role of pericytes in closing/opening capillaries, ask whether their response kinetics are sufficiently fast to support the Q̇ and capillary hemodynamics demonstrated following the onset of contractions? In brain and heart the pericyte-associated constriction is slight and too slow to explain the hyperemia kinetics following contractions onset (c.f. in brain,^208^,^210^,^316^ and heart,^211^ with skeletal muscle^89^). Is there really the possibility for unitary capillary control in muscle, or rather, is fine-tuning of Q̇O_2_-V̇O_2_ matching achieved by ascending vasodilation that increases terminal arteriole diameter and thus RBC flux to all capillaries in a given capillary module.^102,106^,^259–262^ This latter process may be accentuated via venular signaling.^102,106^

The elegant NIRS technology employed by Bowen and colleagues^317^ suggests that temporal Q̇O_2_-V̇O_2_ mismatching in-and-of-itself which leads to a deoxygenation overshoot does not appear to slow V̇O_2_ kinetics; presumably limiting the functional consequences of such fine-tuning of Q̇O_2_-V̇O_2_ matching. Whereas we have supported the case, in health, for Q̇O_2_ kinetics being as fast, or faster, than that of V̇O_2_ (see Myth #6 above) there is evidence during knee-extension exercise that estimated capillary Q̇O_2_ kinetics may be slower (Harper et al.^318^; but see also Schlup et al. ^319^) than that of the femoral artery and this phenomenon is expected to produce a deoxygenation overshoot. Future studies might judiciously account for capillary hemodynamics rather than relying on the tacit presumption that such kinetics are the same as those for arterial Q̇.

Recognizing that strong physiological evidence supports that capillary–mitochondrial diffusion distances do not determine DO_2_. Rather, a key determinant of DO_2_ is the RBC volume of flowing capillaries, which may reflect the importance of newly discovered aquaporin and rhesus pores in the RBC membrane and also the high RBC-capillary endothelium O_2_ flux density.

Understanding more about the role of the endothelial surface layer (glycocalyx) in determining capillary hematocrit and hemodynamics, especially during exercise, in health and perturbations of such in disease. In vivo NIRS measurements during heavy intensity exercise suggest that capillary hematocrit may not reach systemic levels even at very high muscle Q̇s.^120–122^ ^243^

Considering that O_2_ can diffuse between arterioles, capillaries, and venules^136,137,266^ and the presence of higher P*is*O_2_ than (independently) measured for P*im*O_2_ raises the intriguing possibility that O_2_ movement through the interstitial space—around the myocyte periphery and among myocytes—may be important. If so, this provides additional support for the Hill (versus Kroghian “pinpoint”) model of O_2_ diffusion (Figure 5)^107,130^ and this capability would facilitate Q̇O_2_-V̇O_2_ matching irrespective of the marked heterogeneities of capillary RBC flux/hematocrit observed and potentially help explain the hyperbolic rise of O_2_ extraction with increasing V̇O_2_. Of course, it remains to be determined to what extent a higher Q̇O_2_-V̇O_2_ (and thus P*mv*O_2_) in one region might be beneficial if total muscle Q̇O_2_ is constant and such behavior therefore predicates a low P*mv*O_2_ in another region.

Determining the quantitative importance of the multiple pathways for intramyocyte O_2_ transport—aqueous and myoglobin-facilitated O_2_ diffusion and, potentially, that O_2_ tracks along lipid membranes during heavy and severe exercise (Figure 11). This is also true for the role of the mitochondrial reticulum tracking charge potential across the myocyte and the putative role of deoxymyoglobin to enact local NO release thereby controlling cytochrome oxidase activity and protecting ATP production across the myocyte (Figure 8). Development of highly spatially resolved O_2_ sensitive dyes with an active range ∼0.5 to 10 mmHg and rapid response in the subsecond range will be invaluable to this mission.

Incorporating the latest aspects of muscle capillary function models (e.g., Figure 4) into the design and interpretation of investigations. This is especially pertinent, not only in health, but in unveiling the mechanistic bases for dysfunction in diseases such as HF, diabetes, sepsis, and many others.

## Data Availability

No original data are presented herein. Any data previously published by the authors conforms to the individual policy of the relevant journals and, as such, is accessible upon reasonable request to the authors.
